# Identification of a Novel Gene Correlated With Vascular Smooth Muscle Cells Proliferation and Migration in Chronic Thromboembolic Pulmonary Hypertension

**DOI:** 10.3389/fphys.2021.744219

**Published:** 2021-11-11

**Authors:** Feng Wang, Congrui Sun, Xiaoshuo Lv, Mingsheng Sun, Chaozeng Si, Yanan Zhen, Jing Guo, Weiliang Sun, Zhidong Ye, Jianyan Wen, Peng Liu

**Affiliations:** ^1^Department of Cardiovascular Surgery, China-Japan Friendship Hospital, Beijing, China; ^2^Graduate School of Peking Union Medical College, Beijing, China; ^3^Department of Cardiovascular Surgery, Peking University China-Japan Friendship School of Clinical Medicine, Beijing, China; ^4^Department of Operations and Information Management, China-Japan Friendship Hospital, Beijing, China; ^5^Institute of Clinical Medical Sciences, China-Japan Friendship Hospital, Beijing, China

**Keywords:** chronic thromboembolic pulmonary hypertension, vascular remodeling, vascular smooth muscle cells, FOS, TNF-α

## Abstract

**Objective:** Chronic thromboembolic pulmonary hypertension (CTEPH) is characterized by thrombofibrotic obstruction of the proximal pulmonary arteries, which result in vascular remodeling of the distal pulmonary artery. While the cellular and molecular mechanisms underlying CTEPH pathogenesis remain incompletely understood, recent evidence implicates vascular remodeling. Here, we identify the molecular mechanisms that contribute to vascular remodeling in CTEPH.

**Methods:** Microarray data (GSE130391) for patients with CTEPH and healthy controls were downloaded from the Gene Expression Omnibus (GEO) and screened for differentially expressed genes (DEGs). DEGs were functionally annotated using Gene Ontology (GO) functional analysis and Kyoto Encyclopedia of Genes and Genomes (KEGG) pathway analysis. A protein–protein interaction (PPI) network was constructed to identify hub genes. Finally, pulmonary artery samples were harvested from patients with CTEPH (*n* = 10) and from controls (*n* = 10) and primary vascular smooth muscle cells (VSMCs) were cultured. Effects of the proto-oncogene FOS on VSMC proliferation and migration were assessed using expression and knockdown studies.

**Results:** We detected a total of 292 DEGs, including 151 upregulated and 141 downregulated genes. GO analysis revealed enrichment of DEGs in biological processes of signal transduction, response to lipopolysaccharide, signal transduction, and myeloid dendritic cell differentiation. Molecular function analysis revealed enrichment in tumor necrosis factor (TNF)-activated receptor activity, transcriptional activator activity, and protein homodimerization activity. The expression of TNF-α and its receptor (sTNFR1 and sTNFR2) were significantly higher in CTEPH group, compared with control group. KEGG pathway analysis revealed enrichment in salmonella infection, pathways in cancer, osteoclast differentiation, and cytokine-cytokine receptor interaction. Hub genes in the PPI included FOS, suggesting an important role for this gene in vascular remodeling in CTEPH. Primary VSMCs derived from patients with CTEPH showed increased FOS expression and high proliferation and migration, which was attenuated by FOS inhibition. In control VSMCs, TNF-α treatment increased proliferation and migration, which FOS inhibition likewise attenuated.

**Conclusion:** TNF-α drives CTEPH pathogenesis by promoting VSMC proliferation and migration via increased FOS expression. These results advance our understanding of the molecular mechanisms of vascular remodeling in CTEPH, and may inform the development of new therapeutic targets.

## Introduction

Chronic thromboembolic pulmonary hypertension (CTEPH) is a life-threatening condition characterized by an obliteration of proximal pulmonary arteries by intraluminal thrombi and fibrous stenosis (Lang et al., 2013; Toshner and Pepke-Zaba, 2014), which result in vascular remodeling of the distal pulmonary artery. Although patients with CTEPH can experience pulmonary thromboembolism, clinical evidence increasingly suggests that there is no direct relationship between the occurrence and development of pulmonary hypertension and vascular occlusion (Peacock et al., 2006). In fact, the final pathological changes normally associated with CTEPH, including intimal thickening, plexiform lesions, and pulmonary arterial smooth muscle cell (PASMC) proliferation, are similar to the changes in small pulmonary arterial remodeling associated with other types of pulmonary hypertension. Furthermore, changes in PASMC proliferation and migration play an important role in pulmonary arterial remodeling (Tuder et al., 2009; Cirulis et al., 2019) and determine CTEPH progression (Alias et al., 2014; Zhang et al., 2018; Chen et al., 2020). Recent work indicates that the renin-angiotensin system regulates PASMC migration in CTEPH (Zhang et al., 2018) and that EZH2 is highly expressed in CTEPH PASMCs and promotes their migration (Wang et al., 2021). Yet, the cellular events causing pulmonary artery vascular remodeling in CTEPH have not been fully clarified.

Here, we sought to achieve a fuller understanding of CTEPH vascular remodeling using a two-pronged approach. First, we leveraged publicly available microarray data downloaded from the Gene Expression Omnibus (GEO) to screen for all differentially expressed genes (DEGs) between CTEPH and control samples. We used these DEGs in bioinformatic approaches to identify potential differential functions and pathways operating in CTEPH. Second, we evaluated the effects of key genes, tumor necrosis factor (TNF)-α and FOS, on the proliferation and migration of primary vascular smooth muscle cells (VSMCs) taken from patients with CTEPH and from controls. Tumor necrosis factor alpha (TNF-α) is a cytokine with diverse functions. TNF-α signaling occurs via two structurally related receptors, including soluble tumor necrosis factor receptor 1 (sTNFR1) and soluble tumor necrosis factor receptor 2 (sTNFR2) (Corazza et al., 2004). TNF-α has been previously implicated in vascular remodeling (Mattos et al., 2020; Naito et al., 2020; Ogawa et al., 2020). FOS regulates cell proliferation and migration (Zhou et al., 2018) and is upregulated in some cardiovascular diseases (Zhang et al., 2013; Velazquez et al., 2015). Recently study demonstrated FOS was involved in the obliterative pulmonary vascular remodeling (Maron et al., 2014; Patel et al., 2017; Tsujino et al., 2017). However, the roles of TNF-α and FOS in CTEPH were not total clarified. Our results unveil molecular mechanisms underlying CTEPH development and may inform the design both of future experimental studies and of potential treatments.

## Materials and Methods

### Microarray Data

Gene expression data (GSE130391) based on the GPL570 platform were downloaded from the GEO database. The dataset contained 22 tissue samples, including 14 CTEPH samples and four control samples, which were selected for subsequent analysis (the remaining four idiopathic pulmonary hypertension samples were not used).

### Validation of the Dataset

Repeatability between the CTEPH and control groups was evaluated using a Pearson’s correlation test. Correlations between the samples were visualized with a heat map generated using the pheatmap package in R (version 3.6.2). Principal component analysis (PCA) was performed on gene expression profiles to assess sample relationships and variability.

### Identification of Differentially Expressed Genes

The Bioconductor project was used to analyze the high-throughput genomic data, with DEGs between control and CTEPH samples identified using the limma package. A *t*-test was used to calculate DEG *p*-values, with an adjusted *p*-value cut off of 0.05 and the | log FC| > 1.0 as the filter criteria.

### Functional Annotation for Differentially Expressed Genes Using Gene Ontology and Kyoto Encyclopedia of Genes and Genomes Analysis

Gene Ontology and Kyoto Encyclopedia of Genes and Genomes enrichment analyses were performed using the R package clusterProfiler for analyzing and visualizing the functional profiles of genomic coordinates, with the *p*-value and *q*-value cutoffs set to 0.01 and 0.05, respectively.

### Construction and Analysis of the Protein–Protein Interaction Network and Identification of Hub Genes

The online Search Tool for the Retrieval of Interacting Genes (STRING) database was used to predict the PPI network from the imported DEGs. Cytoscape5 was used to visualize the PPI network. The ten genes with the highest Density of Maximum Neighborhood Component (DNMC) score were selected as hub genes using the plug-in cytoHubba.

### Enzyme-Linked Immunosorbent Assay

The concentrations of TNF-α in the serum of two groups were detected by a corresponding enzyme-linked immunosorbent assay (ELISA) kit (SEKH-0047-48T, Solarbio, Beijing, China). After the serums were collected, horseradish peroxidase-labeled detection antibody was added to each well (including standard wells and sample wells), and the serum of each well at a wavelength of 450 nm was measured. The actual concentration of the sample was calculated based on the linear regression curve of the standard sample.

### Immunocytochemistry

Human distal pulmonary artery samples were collected from ten patients undergoing pulmonary endarterectomy (PTE) (CTEPH group) and ten failed donor pulmonary arteries (control group) were derived from lung transplanted donors who died unexpectedly without diseases in the China-Japan Friendship Hospital from October 2017–December 2018, with informed consent obtained according to protocols approved by the hospital’s medical ethics committee. All vascular samples were dehydrated and embedded in paraffin, then sectioned into 5 mm thick slices, dewaxed, rehydrated, and stained with hematoxylin and eosin sequentially until transparent. Sections were then deparaffinated, blocked, and incubated with the primary antibody (FOS, 1:1,000, ab222699, Abcam, United Kingdom; TNF-α, 1:500, 60291-1-lg; sTNFR1,1:200, 21574-1-AP and sTNFR2, 1:200, 19272-1-AP purchased from Proteintech, China) at 4°C overnight. FOS expression was calculated as the integrated optical density (IOD) of the area that was stained yellow-brown using Image-Pro Plus 6.0 (IPP 6.0, Media Cybernetics, United States). IOD was compared across the two groups using an independent *t*-test, with a significance level set at *p* < 0.05.

### Primary Culture of Vascular Smooth Muscle Cells Derived From Normal and Chronic Thromboembolic Pulmonary Hypertension Pulmonary Tissues

Primary VSMCs were prepared from human control and CTEPH pulmonary tissues as previously described (Morris et al., 2010). Briefly, the distal pulmonary artery tissues were cut into small pieces and allowed to attach a plate. Explants were cultured in Dulbecco’s modified Eagle’s medium (DMEM) containing 20% fetal bovine serum (FBS) and incubated at 37°C in an incubator containing 5% CO_2_. VSMCs at passages 2–7 were used for all experiments.

### Immunocytochemistry

The VSMCs were cultured using a chambered cover glass system (Thermo Scientific) for 2 days until they adhered to glass slides. The cells were washed with PBS and fixed with 4% paraformaldehyde for 30 min. After three washes with PBS, the cells were permeabilized with 0.05% Triton X-100 (Sigma) for 20 min and then washed with PBS. The cells were blocked with 3% BSA for 30 min at room temperature and then incubated with α-SMA polyclonal antibody (1:100, 14395-1-AP, Proteintech, China) at 4°C overnight. After washing with PBS, the cells were incubated with secondary antibodies for 2 h at room temperature. The glass slides were mounted with ProLong Gold mounting medium with DAPI (Molecular Probes) and covered with a coverslip. A fluorescence microscope system (Olympus, Japan) was used for imaging.

### Tumor Necrosis Factor Alpha Treatment and FOS Transfection

Normal VSMCs were divided into a control group, a group treated with TNF-α, and a group pretreated with siFOS (siRNA for FOS knockdown) before TNF-α treatment. CTEPH VSMCs were transfected with FOS siRNA or with control siRNA.

### Vascular Smooth Muscle Cell Proliferation Assay

Vascular smooth muscle cells were counted and adjusted to a concentration of 5 × 10^4^ cells/mL. Cell suspension (100 ml) was added to each well of a 96-well plate and incubated at 37°C. VSMCs were treated with 50 ng/mL TNF-α for 24 h and/or with FOS siRNA for 24 h and then incubated at 37°C (Chung et al., 2020). At 4 h (day 0) and days 1, 2, 3, and 4, VSMCs were added to a CCK-8 solution (10 μL/well) and then incubated at 37°C for 30 min. Absorbance was measured with a spectrophotometer at 450 nm. VSMC proliferation was measured using a CCK-8 assay as described previously (Wang et al., 2015).

### Vascular Smooth Muscle Cell Migration Assays

Migration assays included the scratch-wound and transwell assays. For the scratch-wound assay, VSMCs from control group and CTEPH were seeded onto gelatin-coated 6 cm dishes and allowed to form confluent monolayers. VSMCs were mechanically scraped off the bottom of a culture plate with a sterile pipette tip to create a VSMC-free (wounded) area, then washed with medium to remove debris. Wounded cells were maintained in medium and incubated with and without TNF-α (50 ng/mL) for 24 h. The shortest distance between cells that had moved into the wounded region and their respective starting points was determined. Images were acquired for each sample and analyzed using ImageJ software.

For the transwell migration assay, 24-well tissue culture plates with a polycarbonate membrane with 8 μm pores were used (Corning, NY, United States). VSMCs were seeded on the inner chamber of the transwell plate at 1 × 10^5^ cells/100 μL. The inner chamber was placed into the outer chamber filled with 600 μL serum-free DMEM containing TNF-α (50 ng/mL) and incubated at 37°C. The cells that migrated to the outer surface of the membrane were fixed with cold methanol and 4% paraformaldehyde and stained. The number of migrated cells in each sample was counted in 4–6 randomly chosen fields of duplicate chambers at 200× magnification.

### Real-Time PCR

Total RNA in vascular tissues and primary VSMCs was extracted using a Total RNA Extraction Kit (Solarbio; Cat: R1200), following the manufacturer’s protocol. mRNA concentration was assessed using a NanoDrop ND-1000 spectrophotometer. Total RNA was mixed with All-in-One cDNA Synthesis SuperMix (Bimake; Cat: B24403) to generate cDNA. RT-PCR assay was performed with 2 × SYBR Green qPCR Master Mix (Bimake; Cat: B21202) in the LightCycler 480 Real-Time PCR System (RT-PCR, Roche), following the manufacturer’s protocol. The relative expression of targeted genes was calculated using the 2^–ΔΔCt^ method (Nie et al., 2020) and normalized to the expression of β-actin in the VSMCs. The real-time quantitative PCR for TNF-α, FOS, sTNFR1, sTNFR2 and β-actin were carried out using the following primers to amplify genes: TNF-α forward primer, 5′-CTCAAGCCCTGGTATGAGCC-3′ and reverse primer, 5′-TG GACCCAGAGCCACAATTC-3′; FOS forward primer, 5′-CCG GGGATAGCCTCTCTTACT-3′ and reverse primer, 5′-CCAGG TCCGTGCAGAAGTC-3′; sTNFR1 forward primer, 5′-TCACC GCTTCAGAAAACCACC-3′ and reverse primer, 5′-GGTCCA CTGTGCAAGAAGAGA-3′; sTNFR2 forward primer, 5′-TTC ATCCACGGATATTTGCAGG-3′ and reverse primer, 5′-GCTG GGGTAAGTGTACTGCC-3′; β-actin forward primer, 5′-CATG TACGTTGCTATCCAGGC-3′ and reverse primer, 5′-CTCCT TAATGTCACGCACGAT-3′.

### Western Blot

Total protein in the vascular tissues and primary VSMCs was extracted with RIPA Lysis Buffer (Beyotime Technology; Cat: P0013C) containing protease inhibitors and phosphatase inhibitors. Protein concentration was assessed using a BCA Protein Kit (Invitrogen; Cat: 23227), and total protein was separated by a Western blot assay. Equal amounts of each protein sample (30 μg) were separated by sodium dodecyl sulfate-polyacrylamide gel electrophoresis and transferred to polyvinylidene fluoride (PVDF) membranes (GE HealthCare, Marlborough, MA, United States). After blocking the membranes with 5% skim milk in TBST, the membranes were incubated with anti-FOS antibody (1:2,000, 66590-1-lg, Proteintech, China), anti-TNF-α antibody (1:2,000, 60291-1-lg, Proteintech, China), anti-sTNFR1 antibody (1:500, 21574-1-AP, Proteintech, China), anti-sTNFR2 antibody (1:500, 19272-1-AP, Proteintech, China), anti-PCNA antibody (1:2,000, 1025-2-AP, Proteintech, China) and anti-β-actin (1:5,000, 66590-1-lg, Proteintech, China). Then, the membranes were incubated for 1 h at room temperature with an appropriate secondary antibody: horseradish peroxidase (HRP)-conjugated goat anti-rabbit (1:5,000, sc2357, Santa Cruz, United States); anti-mouse (1:5,000, sc516102, Santa Cruz, United States). Bands were detected with Pierce^TM^ ECL Western Blotting Substrate (Thermo Scientific; Cat: 32209).

### Cell Transition Transfection

For FOS transfection experiments, VSMCs were transfected with FOS siRNA or with corresponding control siRNA. The transfection was performed with Lipofectamine 3000 Transfection Reagent (Invitrogen; Cat: L3000015), following the manufacturer’s protocol. FOS expression was detected by Western blot and RT-PCR assays.

### Statistical Analysis

Data are presented as mean ± SD. Differences among normally distributed values of two experimental groups were analyzed by unpaired Student’s *t*-test. Differences of one parameter between normally distributed values of three or more experimental groups were analyzed by one-way ANOVA. *P* < 0.05 was considered statistically significant.

## Results

### Validation of the Dataset

**FIGURE 1:**
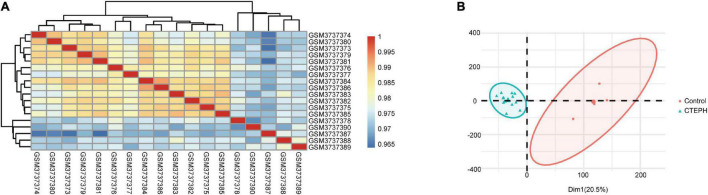
Intra-sample data repeatability test for GSE130391 by Pearson’s correlation analysis and PCA. **(A)** All samples from the GSE130391 dataset were analyzed using Pearson’s correlation analysis. Colors represent the correlation coefficient. **(B)** All samples from the GSE130391 dataset were analyzed using PCA. PC1 and PC2 are represented on the *X*-axis and *Y*-axis, respectively. PCA, principal component analysis; PC1, principal component 1; PC2, principal component 2.

To verify the quality of the GSE130391 dataset and to visualize similarities between the CTEPH and control samples, we constructed a heatmap and conducted PCA. The heatmap revealed strong correlations among the control and CTEPH samples (Figure 1A). PCA showed that the intra-group data repeatability was acceptable. The distances between the CTEPH samples was similar to the distances between control samples for both principal components (PCs) 1 and 2 (Figure 1B).

### Differentially Expressed Genes: Functional, Pathway, and Network Insights

**FIGURE 2:**
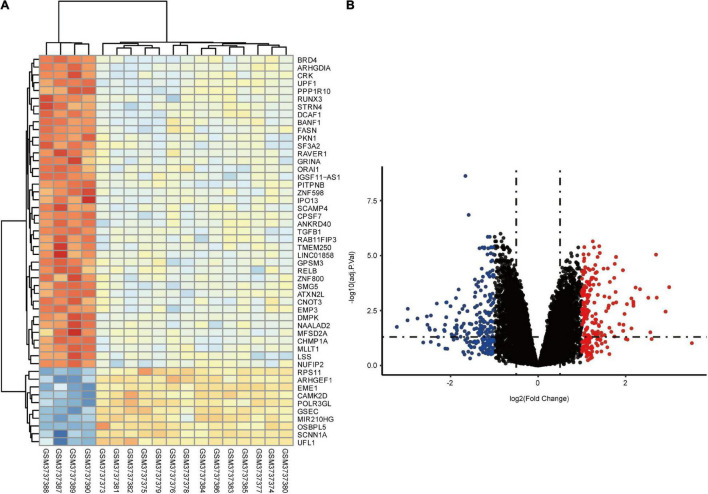
Identification of DEGs between control and CTEPH samples. **(A)** Heatmap showing the DEGs between two groups. **(B)** Volcano plot showing the DEGs between two groups. The *X*-axis represents the | logFC| and the *Y*-axis represents the *p*-value (log-scaled). FC, fold change.

**FIGURE 3:**
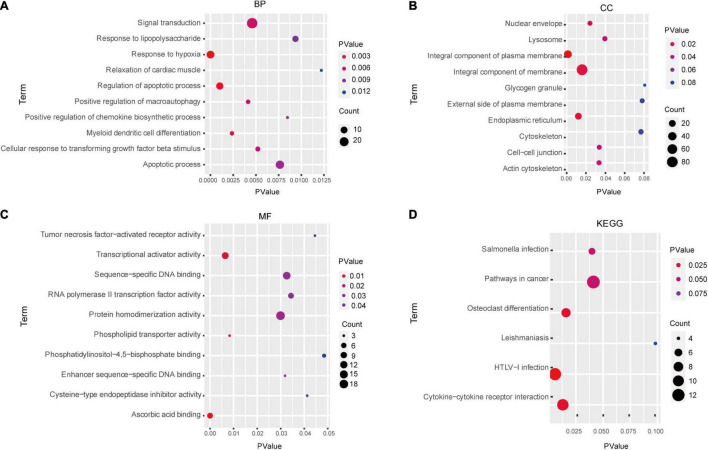
Enrichment analysis of DEGs via GO and KEGG analysis. GO analysis results: **(A)** BP, **(B)** CC, and **(C)** MF. **(D)** KEGG pathways analysis results. BP, biological process; MF, molecular function; CC, cellular component.

**FIGURE 4:**
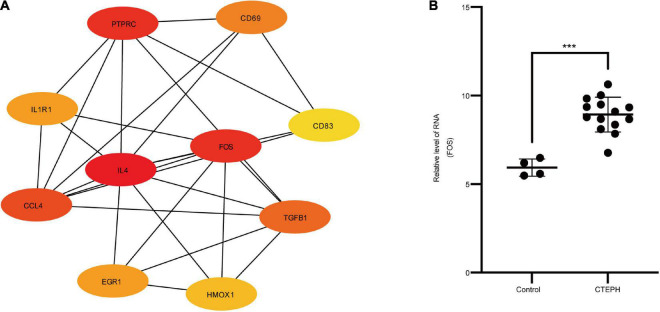
PPI network and hub genes. **(A)** PPI network analyzed by the STRING database. Hub genes included IL4, FOS, PTPRC, CCL4, TGFB1, CD69, EGR1, IL1R1, CD83, and HMOX1. **(B)** The RNA level of FOS in the GSE130391 dataset between control group (*n* = 4) and CTEPH group (*n* = 14). Results are expressed as the mean ± SD, ***p < 0.001. Data were analyzed using student *t*-test.

In total, we identified 292 DEGs among 20,174 genes, including 151 upregulated and 141 downregulated genes. DEGs are illustrated in a heatmap (Figure 2A) and volcano plots (Figure 2B). GO analysis classifies genes into three categories: BP (biological process), MF (molecular function), and CC (cellular component). BP DEGs were enriched in signal transduction, response to lipopolysaccharide, signal transduction, and myeloid dendritic cell differentiation (Figure 3A). CC DEGs were enriched in the nuclear envelope, cell-cell junction, and actin cytoskeleton (Figure 3B). MF DEGs were enriched in tumor necrosis factor (TNF)-activated receptor activity, transcriptional activator activity, and protein homodimerization activity (Figure 3C). KEGG pathways analysis showed that DEGs were mainly enriched in pathways in cancer, osteoclast differentiation and cytokine-cytokine receptor interaction (Figure 3D). In the PPI network, the ten genes with the highest DMNC score were IL4, FOS, PTPRC, CCL4, TGFB1, CD69, EGR1, IL1R1, CD83, and HMOX1 (Figure 4A). FOS is among the top 10 highest expressed genes and the RNA level of FOS is shown in Figure 4B.

### Identification of Vascular Smooth Muscle Cells From Control and Chronic Thromboembolic Pulmonary Hypertension Groups

**FIGURE 5:**
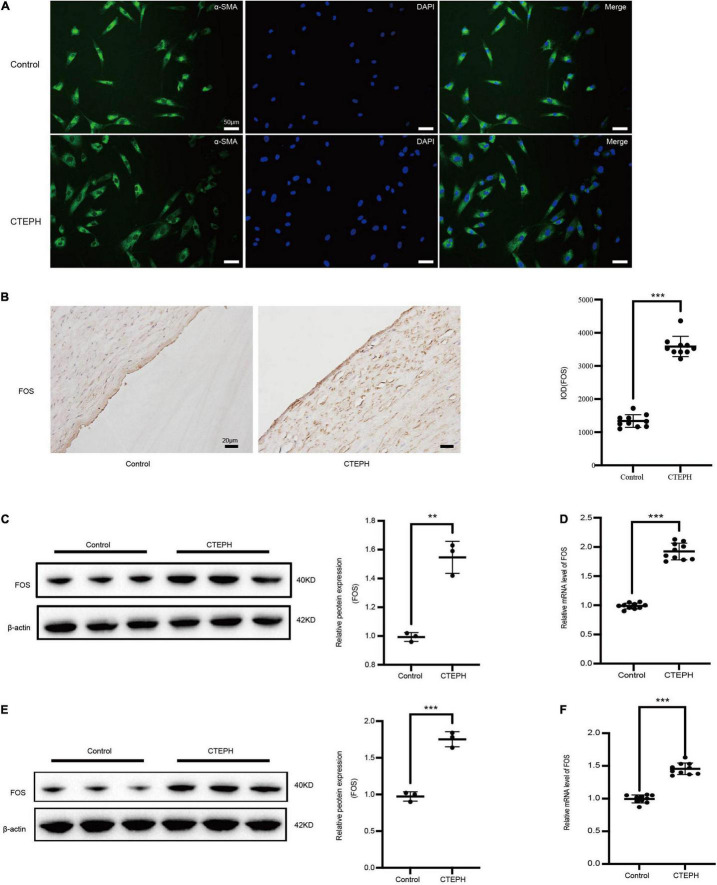
FOS expression in vascular tissues and primarily VSMCs. **(A)** Identification of vascular smooth muscle cells (VSMCs) derived from patients with CTEPH and from controls. Immunocytochemistry analysis shows that the SMCs were positive for alpha smooth muscle actin (α-SMA). **(B)** Representative immunostaining for FOS in control and CTEPH vascular tissue, and corresponding quantification of IOD. *N* = 10, results are expressed as the mean ± SD, ***p < 0.001. FOS protein **(C)** and mRNA **(D)** levels in control and CTEPH vascular tissues and their primary VSMCs **(E,F)** using Western blot and PCR, respectively, *n* = 3 or 10, results are expressed as the mean ± SD, **p < 0.01, and ***p < 0.001. Data were analyzed using student *t*-test.

By immunocytochemistry, the VSMCs expressed the VSMC specific marker α-SMA (Figure 5A).

### FOS Is Highly Expressed Among Human Vascular Tissue and Primary Vascular Smooth Muscle Cells in Chronic Thromboembolic Pulmonary Hypertension

FOS expression in vascular tissues was significantly higher in the CTEPH group than in the control group, as measured by IOD (Figure 5B), Western blot (Figure 5C), and PCR (Figure 5D). Similar results were obtained for FOS expression in primary VSMCs, as measured by Western blot (Figure 5E) and PCR (Figure 5F).

### Increased Expression of Tumor Necrosis Factor Alpha in Chronic Thromboembolic Pulmonary Hypertension Group

**FIGURE 6:**
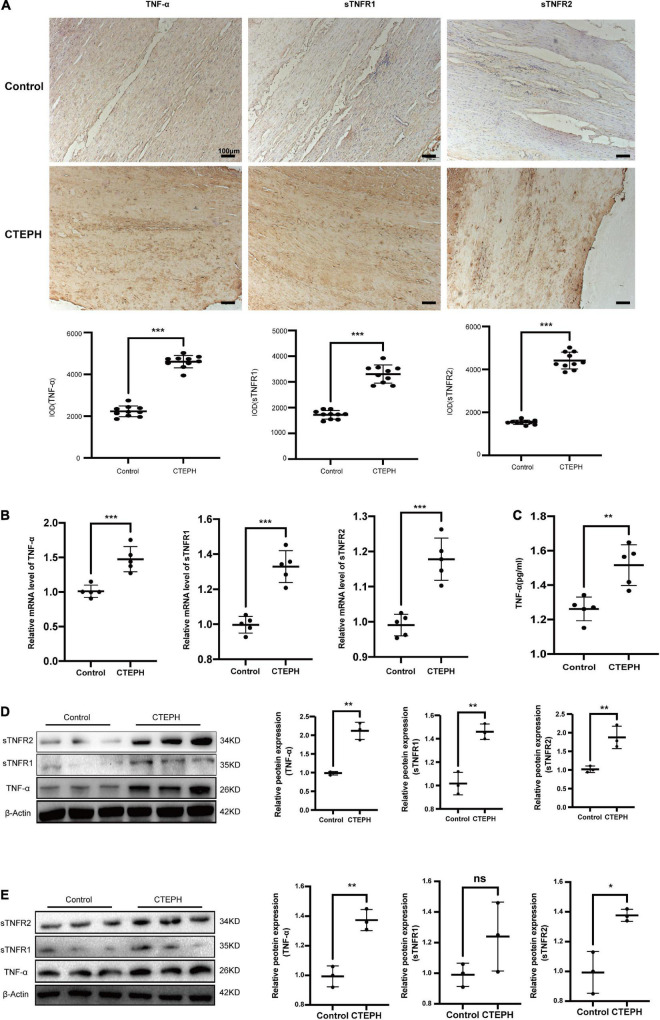
Increased expression of TNF-α in CTEPH group. The expression of TNF-α, sTNFR1 and sTNFR2 were increased in CTEPH group using immunocytochemistry **(A)** and PCR **(B)**, and the corresponding quantitative analysis, *n* = 10 or 5, results are expressed as the mean ± SD, ***p < 0.001. The circulating TNF-α level in the control group and CTEPH group were measured by Elisa assay. The result demonstrated TNF-α level was higher in the CTEPH group comparing with those in the control group. *N* = 5, results are expressed as the mean ± SD, **p < 0.01 **(C)**. The expressions of TNF-α, sTNFR1 and sTNFR2 in vascular tissues and primary VSMCs from two groups measured by Western blot **(D)**. Consistent with the PCR results, the expressions of TNF-α, sTNFR1, and sTNFR2 were significantly higher in CTEPH group compared with control group, *n* = 3, results are expressed as the mean ± SD. ns represent no significantly change, *p < 0.05, **p < 0.01 **(E)**. Data were analyzed using student *t*-test.

To explore the expression of TNF-α and its receptor, we first screened for the changes of TNF-α, sTNFR1 and sTNFR2 using immunocytochemistry and real time PCR. The results revealed that the expressions of TNF-α, sTNFR1 and sTNFR2 were markedly increased in the CTEPH vessel tissues Figures 6A,B. Then we measured the circulating TNF-α level in the control group and CTEPH group by Elisa assay. We found the TNF-α level was higher in the CTEPH group comparing with those in the control group Figure 6C. Lastly, we confirmed the expressions of TNF-α, sTNFR1 and sTNFR2 in vascular tissues and primary VSMCs from two groups by Western blot. Consistent with the PCR results, the expressions of TNF-α, sTNFR1 and sTNFR2 were significantly higher in CTEPH group compared with control group (Figures 6D,E).

### Tumor Necrosis Factor Alpha Stimulates Normal Vascular Smooth Muscle Cell Proliferation and Migration via Increased FOS Expression

**FIGURE 7:**
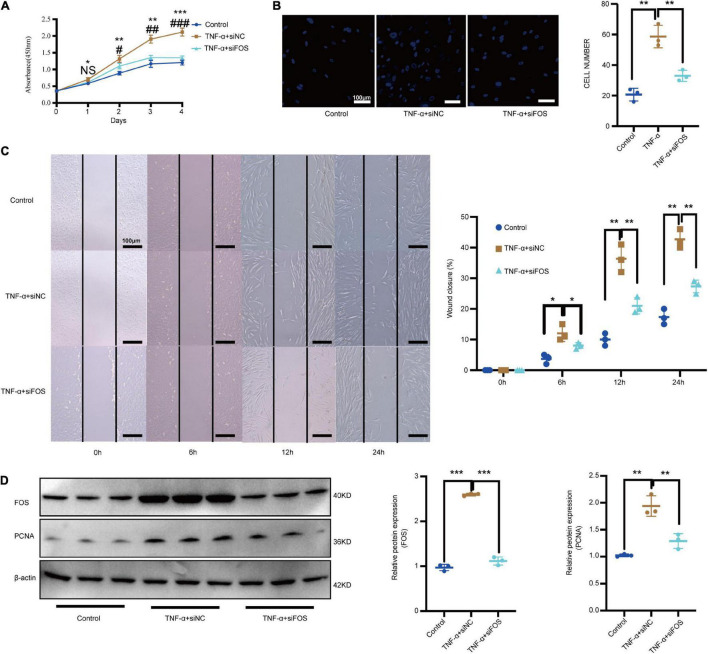
TNF-α promotes the proliferation and migration of normal VSMCs via increased FOS expression. Proliferation and migration of normal VSMCs as measured by **(A)** CCK-8 assay, **(B)** transwell assay, and **(C)** scratch-wound assay after treatment with TNF-α or TNF-α treatment (50 ng/mL, 24 h) plus preincubation with FOS-siRNA (24 h). **(D)** Western blot analysis of PCNA and FOS in normal VSMCs after treatment with TNF-α or TNF-α treatment (50 ng/mL, 24 h) plus preincubation with FOS-siRNA (24 h). *N* = 3, results are expressed as the mean ± SD. *represent TNF-α + siNC VS Control; ^#^represent TNF-α + siFOS VS TNF-α + siNC *p < 0.05, **p < 0.01, and ***p < 0.001. NS represent no significantly change, ^#^*p* < 0.05, ^##^*p* < 0.01, and ^###^*p* < 0.001. Data were analyzed using one-way ANOVA.

TNF-α plays important regulatory roles in mediating the VSMC proliferation and migration response to an array of stimuli (Chung et al., 2020; Naito et al., 2020). Meanwhile, our GO results showed that MF DEGs were enriched in the TNF-α signaling pathway. To determine whether TNF-α can alter VSMC proliferation and migration via increased FOS expression *in vitro*, we compared proliferation and migration in control VSMCs treated with and without TNF-α. TNF-α treatment significantly increased proliferation, as measured by a CCK-8 assay (Figure 7A), and migration, as measured by transwell assays and scratch-wound (Figures 7B,C). TNF-α also increased the protein levels of FOS and proliferating cell nuclear antigen (PCNA), as measured by Western blot (Figure 7D). To study the effects of FOS on proliferation and migration, we knocked down FOS in control VSMCs using siRNA before NF-α treatment. Pretreatment with FOS siRNA significantly attenuated the effect of TNF-α on proliferation and migration. These results suggest that TNF-α promotes FOS upregulation and induces proliferation and migration in normal VSMCs.

### Inhibition of FOS Attenuates Proliferation and Migration of Chronic Thromboembolic Pulmonary Hypertension Vascular Smooth Muscle Cells

**FIGURE 8:**
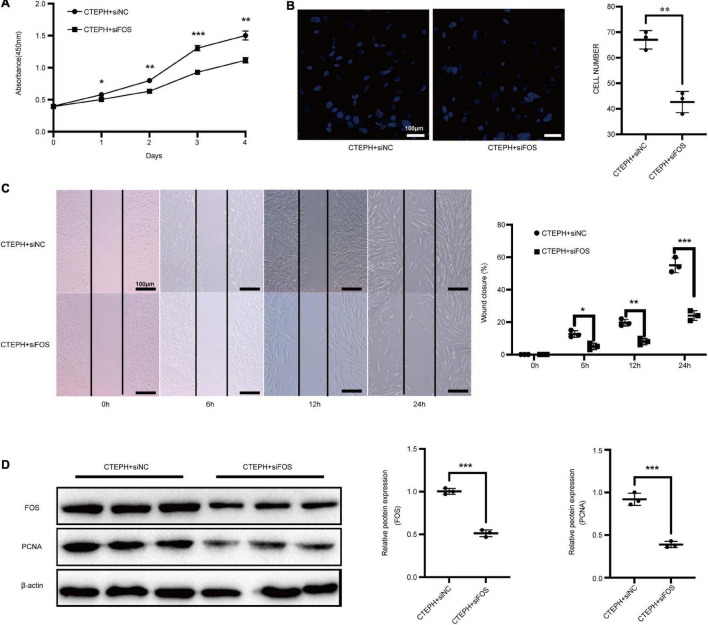
Inhibition of FOS attenuates the proliferation and migration of VSMCs in CTEPH. Proliferation and migration of CTEPH VSMCs as measured by **(A)** CCK-8 assay, **(B)** transwell assay, and **(C)** scratch-wound assay after treatment with FOS-siRNA for 24 h. **(D)** Western blot analysis of FOS and PCNA in CTEPH VSMCs after treatment with FOS-siRNA for 24 h. *N* = 3, results are expressed as the mean ± SD, *p < 0.05, **p < 0.01, and ***p < 0.001. Data were analyzed using student *t*-test.

We previously showed that CTEPH VSMCs exhibit higher proliferation and migration abilities than normal VSMCs (Zhang et al., 2018). To examine whether FOS overexpression facilitates VSMC proliferation and migration, we used siRNA to knock down FOS in CTEPH VSMCs. Proliferation was significantly reduced in CTEPH VSMCs transfected with FOS siRNA vs. with control siRNA, as measured by a CCK-8 assay (Figure 8A). FOS knockdown abolished the migration ability of CTEPH VSMCs, as shown in the transwell assays and scratch-wound (Figures 8B,C). FOS and PCNA expression levels in CTEPH VSMCs decreased after FOS knockdown (Figure 8D). These results indicate that FOS may play a regulatory role in the vascular remodeling in CTEPH by promoting VSMC proliferation and migration.

## Discussion

Chronic thromboembolic pulmonary hypertension is one of the most serious complications of acute pulmonary embolism (Delcroix et al., 2013; Deng et al., 2016; Stam et al., 2018). Reversing the distal pulmonary artery remodeling that characterizes this condition might improve both hypoxemia and residual pulmonary hypertension (PAH) and is a goal for future CTEPH treatment (Mandras et al., 2020). However, strict case selection and surgeon experience are critical features in determining the success of surgery. Despite the use of PAH-specific drugs to modulate the increased pulmonary vascular pressure (Galie et al., 2015), survival among patients with CTEPH remains poor, with mean 5-year survival rates of 53–69% (Radegran et al., 2016; Boucly et al., 2017; Quadery et al., 2018). Further insight into the complex pathogenesis of CTEPH is therefore urgently needed. In this study, we detected 292 significant DEGs in the CTEPH vs. control group, including 151 up- and 141 down-regulated genes. GO analysis showed that these DEGs were mainly associated with TNF-α-activated receptor activity, signal transduction, and myeloid dendritic cell differentiation, while KEGG pathway analysis revealed pathways in cancer, osteoclast differentiation, and cytokine-cytokine receptor interaction.

TNF-α signaling occurs via two structurally related but functionally distinct receptors, sTNFR1 proinflammatory effects whereas sTNFR2 is related to tissue repair, growth-modulating and differentiation (Ihnatko and Kubes, 2007; Gaida et al., 2012). The association between vascular remodeling and TNF-α activation and cell apoptosis is well established. Serum TNF-α levels are high in patients with CTEPH (Naito et al., 2020). VSMC proliferation and migration is promoted by TNF-α in other diseases (Chung et al., 2020; Fan et al., 2021; Zhu et al., 2021), and by TNF-α and other transcription factors in some cardiovascular diseases (Fan et al., 2021; Li et al., 2021; Xu et al., 2021). TNF-α expression is correlated with high expression of tissue factor (TF), and may be useful in determining the prognosis of patients with CTEPH (Yang et al., 2016). The finding that the renin-angiotensin system regulates pulmonary arterial smooth muscle cell migration may prove beneficial in the development of novel therapies for CTEPH (Zhang et al., 2018). MicroRNA let-7d promote PASMCs proliferation and may also be involved in the pathogenesis of CTEPH (Wang et al., 2013). However, whether TNF-α contributes to vascular remodeling via regulation of VSMC proliferation and migration was unclear. Here, we found TNF-α and its receptors were significantly higher in vascular tissues of CTEPH group, while the sTNFR1 has no significant change in primary VSMCs comparing with control group. These results demonstrated inflammation and VSMCs proliferation may play an important pathogenesis of CTEPH progress. Meanwhile, the results showed that TNF-α significantly increased the proliferation and migration abilities of normal VSMCs.

To explore the molecular mechanisms underlying TNF-α’s effects, we constructed a PPI network. Among the top ten hub genes identified (IL4, FOS, PTPRC, CCL4, TGFB1, CD69, EGR1, IL1R1, CD83, and HMOX1), FOS was involved in the TNF-α signaling pathway and was significantly upregulated in CTEPH VSMCs. FOS, considered an immediate early gene because of its transient and rapid change in expression in response to stimuli, participates broadly in the regulation of cell proliferation and migration (Chen et al., 2016; van et al., 2017; Alhendi et al., 2018). FOS upregulation is observed in some cardiovascular diseases, including viral myocarditis, acute myocardial infarction, heart failure, and abdominal aortic aneurysms (Zhang et al., 2013; Zhao et al., 2019), indicating a potential link between FOS and these pathophysiologic processes. Meanwhile, more and more study demonstrated FOS was involved in the obliterative pulmonary vascular remodeling (Maron et al., 2014; Patel et al., 2017; Tsujino et al., 2017). These results demonstrated FOS may play a vital role in the pulmonary vascular remodeling, which provided a new perspective to study the pathophysiological process of CTEPH. To date, however, it was not clear whether FOS could drive CTEPH pathogenesis by altering the vascular remodeling or the biological properties of VSMCs. We found that FOS mRNA and protein levels were significantly increased in both the vascular tissues and their primary VSMCs taken from patients with CTEPH, compared to controls. FOS inhibition by siRNA attenuated the proliferation and migration of VSMC stimulated by TNF-α. These results demonstrate the interactive effect between TNF-α and FOS in regulating VSMC proliferation and migration. They are also consistent with previous work showing that increased FOS expression mediates TNF-α’s promotion of VSMC migration during pulmonary hypertension in rats (Hiram et al., 2015).

Our study has several limitations. First, the relatively small sample sizes in the GEO130391 dataset (14 CTEPH vs. four control) may limit power. Second, FOS expression could have been altered during surgical harvesting and manual distension. FOS protein expression can also be activated in patients with cancer, and while we carefully excluded patients with a history of cancer, it is possible that some participants had had undiscovered cancer. Third, we isolated only primary VSMCs, and not endothelial cells, from the vascular samples. We aim to analyze different cell types in a future study.

## Conclusion

Our study demonstrates that TNF-α participates in the pathogenesis of CTEPH by promoting the proliferation and migration of VSMCs via increased FOS expression. These results suggest that the TNF-α pathway may play a crucial role in vascular remodeling in CTEPH. They also identify FOS and TNF-α as potential therapeutic targets for the prevention of vascular remodeling. We showed clear transcriptomic differences between control and CTEPH vascular tissue, with FOS and PCNA for example being upregulated in CTEPH samples. Characterization of these RNAs may provide new targets for understanding CTEPH diagnosis, progression and treatment.

## Data Availability Statement

The raw data supporting the conclusions of this article will be made available by the authors, without undue reservation.

## Ethics Statement

The studies involving human participants were reviewed and approved by the Ethics Committee of China-Japan Friendship Hospital. The patients/participants provided their written informed consent to participate in this study.

## Author Contributions

JW and PL designed, guided, and funded the study. FW conducted most of the experimental work. CSi helped with the software calculation. FW, CSu, XL, and MS performed the data analysis, immunocytochemistry, and VSMC assays. FW and JW drafted the manuscript. JW and PL critically revised the manuscript. All authors contributed to the article and approved the submitted version.

## Conflict of Interest

The authors declare that the research was conducted in the absence of any commercial or financial relationships that could be construed as a potential conflict of interest.

## Publisher’s Note

All claims expressed in this article are solely those of the authors and do not necessarily represent those of their affiliated organizations, or those of the publisher, the editors and the reviewers. Any product that may be evaluated in this article, or claim that may be made by its manufacturer, is not guaranteed or endorsed by the publisher.
